# Oscillation Encoding of Individual Differences in Speech Perception

**DOI:** 10.1371/journal.pone.0100901

**Published:** 2014-07-03

**Authors:** Yu Jin, Begoña Díaz, Marc Colomer, Núria Sebastián-Gallés

**Affiliations:** Speech Acquisition and Perception Group, Center for Brain and Cognition, Department of Technology, Pompeu Fabra University, Barcelona, Spain; University of Salamanca- Institute for Neuroscience of Castille and Leon and Medical School, Spain

## Abstract

Individual differences in second language (L2) phoneme perception (within the normal population) have been related to speech perception abilities, also observed in the native language, in studies assessing the electrophysiological response mismatch negativity (MMN). Here, we investigate the brain oscillatory dynamics in the theta band, the spectral correlate of the MMN, that underpin success in phoneme learning. Using previous data obtained in an MMN paradigm, the dynamics of cortical oscillations while perceiving native and unknown phonemes and nonlinguistic stimuli were studied in two groups of participants classified as good and poor perceivers (GPs and PPs), according to their L2 phoneme discrimination abilities. The results showed that for GPs, as compared to PPs, processing of a native phoneme change produced a significant increase in theta power. Stimulus time-locked analysis event-related spectral perturbation (ERSP) showed differences for the theta band within the MMN time window (between 70 and 240 ms) for the native deviant phoneme. No other significant difference between the two groups was observed for the other phoneme or nonlinguistic stimuli. The dynamic patterns in the theta-band may reflect early automatic change detection for familiar speech sounds in the brain. The behavioral differences between the two groups may reflect individual variations in activating brain circuits at a perceptual level.

## Introduction

A particularly challenging theoretical question in the field of language learning is addressing the large individual differences in second language (L2) mastery. What makes some people more successful non-native language learners than others? Previous research has identified different factors involved in successful learning, such as age of acquisition, amount of previous experience, working memory, attention control, or motivation [1]–[6]. But even when controlling for all of these variables, substantial individual differences persist, in particular in the perception and production of speech sounds. With the advent of new neurophysiological and imaging methods, the inquiry into individual differences in second language learning has moved to a new level of analysis in terms of how individual brains work [7]–[14]. One attractive feature of some neural-based methods is the possibility of directly measuring the brain activity, removing the need to ask participants for overt responses and eliminating response-related effects. One of the most widely used measures of second-language speech perception is the event-related response (ERP) mismatch negativity (MMN) that is measured during passive listening and signals auditory discrimination sensitivity. The MMN has been showed to capture differences in individual phoneme discrimination capabilities in healthy populations [7], [15]. The present study investigates the oscillatory neural patterns related to success in phoneme learning by analyzing the spectral dynamics underneath the MMN responses of individuals with different levels of mastery of L2 phonemes.

The MMN is elicited by “deviant” sounds; these are sounds that violate the preceding sound sequence. The MMN is elicited without participants’ awareness [16] and even when attending to an unrelated task to the auditory stimulation [17]. The MMN system is considered to operate preattentively. However, the elicitation of MMN *per se* does not imply that all processes leading to the detection of deviants are also attention independent [18], [19]. The MMN peaks between 100–250 ms after the auditory change, with a negative fronto-central scalp distribution. The main neural source of the MMN has been located in the supratemporal plane, in or near the primary auditory cortex, with additional contributions from the frontal and parietal lobes [20]–[31]. The MMN has been proved to be a very useful tool for investigating different aspects of speech perception in normal and pathological populations [32]–[36]. Relevant to our current goals, the amplitude of the MMN is directly related to the magnitude of the perceived change and, hence, it is considered a measure of individual auditory discrimination accuracy [37], [38].

Differences in MMN amplitude are used to characterize individual differences in speech perception. [7] compared two groups of highly skilled bilinguals (Good Perceivers, GPs, and Poor Perceivers, PPs) who differed in their capacity to perceive an L2 vowel contrast. The classification was performed based on their performance on different behavioral tasks [39]. For the two groups of participants, we recorded ERPs responses to nonlinguistic (perception of frequency, duration, and presentation order differences in tones) and speech (perception of spectral frequency differences in native and unknown vowels) changes. Importantly, the unknown vowel did not belong to participants’ L2. The results showed larger MMNs over frontal electrodes for GPs when compared to PPs, only for speech sounds, native and unknown. Moreover, the difference in MMN amplitude between the groups was present at the frontal electrodes, but absent at the supratemporal ones. The absence of differences in the acoustic conditions indicated that the perceptual analysis of simple sound features and their neural memory representation were not the cause of the behavioral differences between the GPs and PPs. This indicates that the origin of individual variability in L2 phoneme mastery is rather speech-specific. Furthermore, the similarity of responses in the acoustic conditions (and also at the temporal electrodes for the speech conditions) ruled out any account based on general attention differences between the two groups. In an ERP study testing unknown phonetic contrast (neither L1 nor L2) discrimination abilities in successful versus unsuccessful L2 learners, [15] reported analogous findings. They concluded that unsuccessful L2 learners have a less efficient speech process than successful L2 learners. Together, these findings in different populations suggest a speech-related origin (rather than a perceptual one) underlying individual differences in speech perception.

Although by using EEG signals solely it is not possible to infer the exact location and nature of the neural contributions underlying the MMN, EEG recordings in lesion patients [40], source imaging (EEG combined with fMRI or MEG, [41], [42]), dipole sources modelling with EEG [29], [43], [44] or new approaches to EEG signal analysis, like Independent Component Analysis (ICA, [27]) have consistently revealed the disassociated functions of frontal and temporal MMN generators. These methods have allowed researchers to infer that the temporal MMN generator is closely associated with integrating information from the sensory input streams with memory traces, whereas the frontal (and parietal) generator is in part related to an involuntary attention-switching mechanism responsible for the detection of deviant sounds. Since GPs and PPs differed at frontal electrodes during speech discrimination, whereas no differences were found at temporal sites, [7] concluded that the two groups differed in their attention orienting mechanism involved in speech change-detection. Yet, EEG signals are thought to be the summation of oscillatory activities and reliance on measures of peak amplitude calculated from an average waveform, as the MMN, have a limitation – they could be hiding the underlying oscillatory mechanisms involved in the EEG generation. Therefore, the lack of differences in the MMN amplitude for the speech changes at the temporal electrodes between GPs and PPs could uncover potential differences in the oscillatory modulations at temporal sites. Following the same rationale, oscillatory differences between GPs and PPs during the processing of nonlinguistic changes may not be captured by the MMN. The present study aims to examine the underlying oscillatory responses during the MMN, and whether they contribute to the observed individual differences. This will be assessed by comparing GPs’ and PPs’ oscillatory responses underlying the MMN responses to nonlinguistic and speech changes.

Several EEG and magnetoencephalography (MEG) studies suggested that the auditory discriminatory process reflected by the MMN is accompanied by phase alignment and power modulation at the theta frequency range [45]–[48]. Besides auditory discrimination, the theta band is associated to several cognitive functions as working memory processes, attentional processing, spatial navigation and (episodic) long-term memory processes [49]. [45] used time-frequency analysis of single-trial ERPs to demonstrate that the MMN is due to a combination of increased theta power and phase alignment for deviant trials. They also found that amplitude modulation and phase alignment mechanisms depend on the source location of the MMN: event-related spectral modulation was higher for deviants than for standards at frontal, but not at temporal sites. [46] revealed a similar finding in an MEG study: the phase modulation of theta oscillation during a passive oddball paradigm was associated with deviant evoked responses. They identified phase synchronizations between temporal and ipsilateral frontal regions, as well as temporal-temporal and temporal-parietal synchronies. [47] performed single trial analyses of the MMN (subtracting deviant trials from the preceding standard). They found no evidence for event-related spectral power changes, but there was a significant event-related phase alignment in the theta frequency. Relevant for the topic of individual differences, the phase alignment in the theta band was a predictor of behavioral discriminability of a difficult acoustic (i.e. frequency) change. All these previous studies indicate that the MMN response is related to theta power and phase modulations. [48] addressed the question of whether the EEG oscillations underlying the MMN are elicited by acoustic stimuli and/or by the presentation probability, since the MMN is usually measured in oddball paradigms, during which infrequent deviant sounds violate an auditory regularity engendered by frequently presented standard sounds. To eliminate the effect of probability differences, the neural response to the same sounds was compared when presented in an oddball paradigm and in a control paradigm in which the tones were presented with equal probability. In the oddball paradigm, an ERSP and ITC increase in the theta band was associated with the presentation of the deviant stimuli, whereas no significant event-related spectral power changes were detected for the control paradigm. Their results were in broad agreement with previous studies showing that the MMN response in the oddball paradigm is related to theta power and phase modulations. Additionally, this study proved that the oscillatory changes in theta are caused by the violation of auditory regularities, rather than by acoustic changes alone. Based on these findings, we expect that the MMN differences related to phoneme discrimination reported by [7] will be accompanied by differences in spectral modulations in the theta band.

Here, we assessed the differences between GPs and PPs using measures of EEG event-related spectral power (ERSP) and intertrial coherence (ITC). ERSP measures spectral power changes at a given frequency range, time-locked to a stimulus event relative to pre-stimulus baseline. ITC measures the extent to which activity at a given frequency is in phase across different trials in time, and is indicative of event-related phase resetting. Furthermore, we will study the ERSP and ITC changes associated with deviant and standard sounds separately. Taken together the reported involvement of the theta rhythm in the MMN [45]–[48] and our previous data on individual differences in speech sound perception [7], we hypothesized that differences between GPs and PPs for vowel processing are most likely related to the modulations (amplitude and/or phase-locking) of theta frequency oscillations (4–8 Hz) during the speech conditions, particularly in frontal areas, whereas no differences were expected for the processing of nonlinguistic stimuli.

## Materials and Methods

### EEG Data Acquisition

We applied EEG spectrum analyses on the same EEG data set studied in [7]. Here, we will describe briefly the data collection procedure (for a detailed description, see [7]). In that study, a relationship between native and non-native phoneme perception capacities was reported in healthy adults. Thus, the researchers first selected two groups of early and highly proficient Spanish (first language, L1) - Catalan (second language, L2) bilinguals differing in their capacity to perceive a difficult vowel contrast in their L2 (the mid-front Catalan vowel contrast/e/-/ε/). Sixteen participants were considered good perceivers (GPs) because they scored within the range of natives in three behavioral tasks (a phoneme discrimination task, a gating task and an auditory lexical decision task; see [39] for details). Fifteen participants were considered poor perceivers (PPs) because they did not score within the range of natives in any of the three tasks. The two groups represented exceptionally good and poor L2 perceivers, as approximately, 23% of the original sample of 126 participants was classified as PPs and 12% as GPs. In [7] the data from one PP was excluded because there were not enough EEG epochs free of artifacts (<70%). Following the same exclusion criteria, one GP participant was excluded in the frequency condition and one additional PP participant was excluded in the duration condition. In the present study the same participants as in [7] were included in each condition.

Participantś central sound representation was measured for conditions tapping general acoustic perception (duration, frequency, and pattern conditions) and speech perception (native and nonnative phoneme conditions). During the EEG recording, participants were asked to watch a silent movie and to ignore the auditory stimulation.

In the duration condition, the stimuli were four pure tones of 1,000 Hz: the standard tone was 200 ms, and the three deviant tones were 120, 80 and 40 ms. In the frequency condition, stimuli were four pure tones of 50 ms: the standard tone was 1,000 Hz, and the deviant tones were 1,030, 1,060, and 1,090 Hz. In both conditions the presentation probability was 0.8 (1,200 presentations) for the standard and 0.066 (100 presentations) for each deviant. Tones were presented in random order with the restriction that at least one standard tone was presented between two deviants. The stimulus onset asynchrony (SOA) was of 314 ms. In the pattern condition, 400 trains of 50 ms-tones were presented. Each train consisted of six alternating pure tones of either 500 or 1,000 Hz (2,400 tones altogether). Tones were presented at a SOA of 128 ms. Stimulus trains were presented in a predictable way (ABABAB-BABABA-**B**ABABA-ABABAB…), in which A represents the 500 Hz tone and B the 1,000 Hz tone, the hyphen indicates the beginning of the trains, and A and B denote the deviant event (i.e., repetition of the last tone presented in the preceding train).

In the native and unknown phoneme conditions, the same synthesized phonemes used by [35] were presented. The standard stimulus for both native and unknown phoneme conditions was the Spanish vowel/o/with a presentation probability of 0.8. The native deviant phoneme was the Spanish vowel/e/and the unknown deviant phoneme was the Estonian vowel/ö/ (unfamiliar to all participants), with a presentation probability of 0.2 each. As described in [7], the acoustic properties of the Finnish/e/and/o/vowels employed by [35] were similar to the Spanish/e/and/o/vowels. Both native and unknown phoneme blocks contained 500 stimuli each (400 standards and 100 deviants) with a constant stimulus onset asynchrony (SOA) of 488 ms. The duration of all the phonemes was 200 ms. The stimuli were presented at random but there was at least one standard stimulus before a deviant one.

### EEG Data Processing

To investigate the neural oscillatory changes associated to the MMN, we applied spectral analyses on EEG data to measure event-related spectral perturbation (ERSP) and intertrial coherence (ITC) for those stimuli that elicited a MMN in [7]: for the duration condition the standard 200 ms-tone vs. the deviant 40 ms-tone (40 ms), for the frequency condition the standard 1,000 Hz-tone vs. the deviant 1,090 Hz-tone, in the pattern condition the standard alternating tones vs. the repeated tones, in the native phoneme condition the standard/o/vs. the deviant/e/, and in the nonnative phoneme condition the standard/o/vs. the deviant/ö/. The EEG data processing is detailed below, as it is different from the previous study [7].

The EEG data was digitized at 500 Hz and band-pass filtered (0.01 to 80 Hz). A 50 Hz notch filter was employed. Eye blinks and other focal artifacts were removed using independent component analysis (ICA) implemented in BrainVision Analyzer software (Brain Products GmbH, Munich, Germany). The data was segmented into 2000 ms epochs, including a pre-stimulus baseline of 500 milliseconds. The epochs were sorted into standard and deviant trials. For all the standard epochs, there were no deviant stimuli presented in the 2000 ms window. Because deviant stimuli were always preceded and followed by standard stimuli, the deviant epochs included the presentation of standard stimuli. In the deviant epochs no other deviant was presented. Therefore, the standard and deviant epochs only differed in the stimuli presented at time 0. The scalp electrode positions included in the analysis were: C3, C4, Cz, F3, F4, F5, F6, F7, F8, Fz, LM, P3, P4, Pz, RM, T3L, and T4L. The EEG spectrum analyses were performed using the EEGLAB software [50].

### ERSP and ITC

ERSP and ITC were computed on the individual 2s-epochs using the *newtimef* function of EEGLAB. Spectral decompositions were done from 0 to 50 Hz using Morlet wavelets with a constant 1 cycle length.

ERSP measures average dynamic changes in the amplitude of the EEG frequency spectrum as a function of time relative to the onset of the experimental stimulus. In the current study, ERSP values (dB) were computed using a 500 ms time window relative to a 200 ms baseline period.

The ITC (*newtimef* function) is a measure of consistency of the EEG spectral phase at different frequency ranges and times across epochs. ITC values range from 0 to 1, with values near 1 implying almost perfect phase coincidence across epochs. In the present study, ITC values were computed for a 0–500 ms time window.

### Statistical analysis

For the ERSP and ITC statistical analyses we combined the use of time-frequency analysis with more conventional amplitude criteria for identifying periods of significant changes in ERSP and ITC. We compared GPs and PPs for standard and deviant trials separately for the frequency bands theta (4–8 Hz), alpha (8–12 Hz), beta (12–30 Hz), and gamma (30–50 Hz). To control for multiple testing of data points at each electrode, we required a minimum sequence length of 8 consecutive data points (56 ms) to exceed the significance level (p<0.05) for an interval of 200 ms [47], [51].

### Ethics Statement

The experiment was approved by the local ethical committee of the University of Barcelona and it was in compliance with the Code of Ethics of the World Medical Association (Declaration of Helsinki). Written consent was obtained from each participant prior to the experiment. All participants were paid at the end of the experiment for their participation.

## Results

The epoch numbers for each condition and stimulus type were not different between the two groups (Table 1), yet there was a trend towards PPs having more epochs than GPs for the phoneme stimuli. Since spectral analyses may depend on the number of epochs, a sub-set of epochs for the PPs that matched the number of segments for GPs were randomly selected to be analyzed for the phoneme stimuli.

**Table 1:** Epoch numbers in the different conditions, for the GPs and PPs groups.

	PPs	GPs	t(df), p value
Native phoneme standards	156.86±15.84*	126±4.75	t(28) = 1.97, p = 0.06
Native phoneme deviants	85.29±8.15*	72.94±2.88	t(28) = 1.50, p = 0.15
Unknown phoneme standards	156.71±13.98*	130.94±3.30	t(28) = 1.91, p = 0.07
Unknown phoneme deviants	89.14±7.63*	76.13±1.91	t(28) = 1.76, p = 0.09
Frequency standards	727.57±20.34	731.93±52.26	t(27)<1, p = 0.77
Frequency deviants	98.64±1.82	98.86±6.24	t(27)<1, p = 0.89
Duration standards	749.84±108.87	722.93±36.26	t(27)<1, p = 0.37
Duration deviants	102.69±14.48	98.62±6.50	t(27) = 1.01, p = 0.35
Pattern standards	415.57±54.44	397.12±6.25	t(28) = 1.34, p = 0.22
Pattern deviants	415.71±54.68	397.18±6.30	t(28) = 1.34, p = 0.21

*For the spectral analysis, the number of segments for the PPs was randomly selected to match the number of segments for the GPs: 135.35±7.11 native phoneme standards, 77.35±6.14 native phoneme deviants, 136.14±10.02 unknown phoneme standards and 79.42±4.5 unknown phoneme deviants. There were no differences between the groups in the number of segments for any phoneme stimulus (for all t-tests t<1).

### ERSP

**Figure 1:**
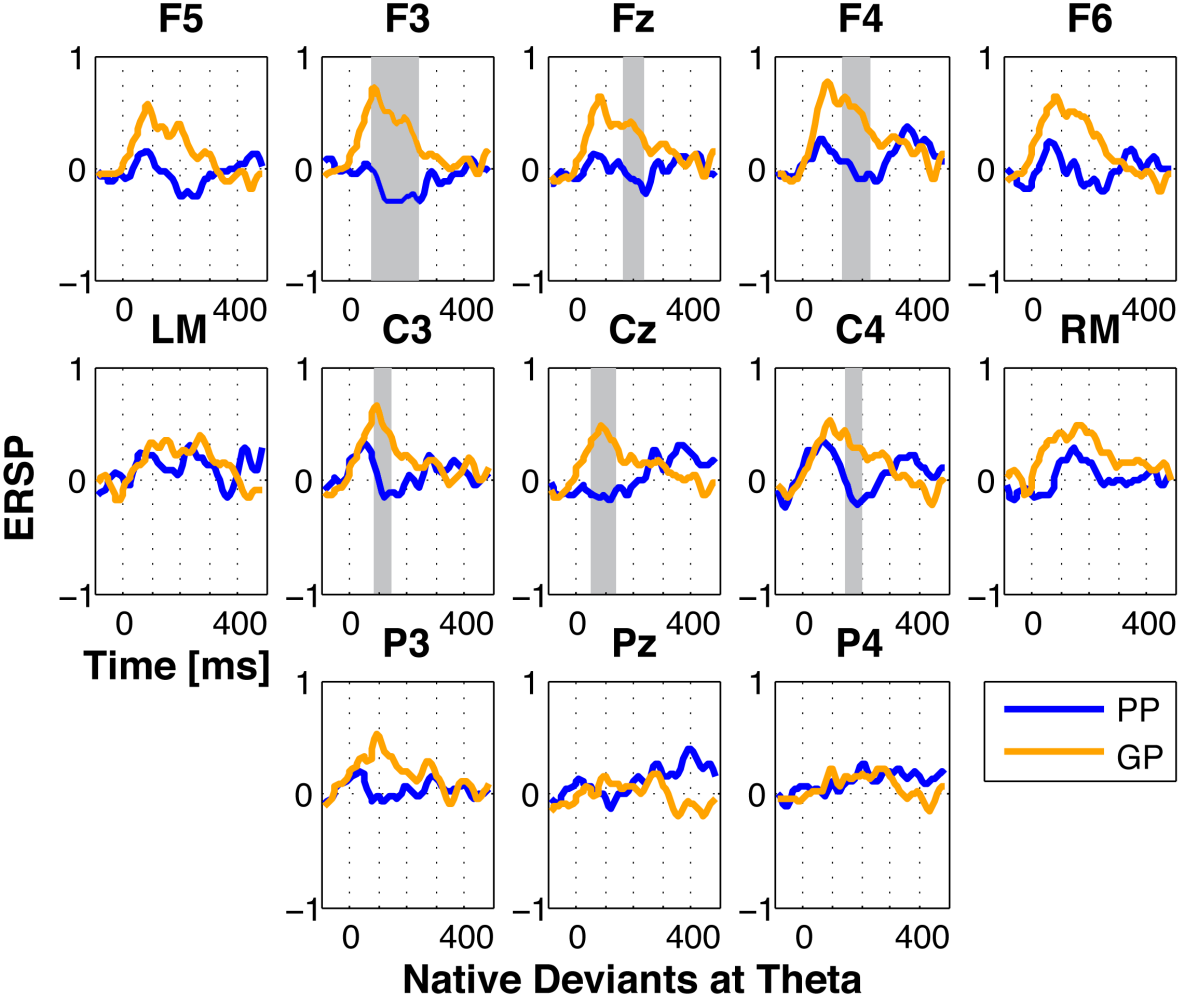
ERSP for the theta band time-locked to the onset of the native deviant phoneme. The grey bars depict the time windows where t-tests yielded significant differences (i.e., p<0.05 at least for eight consecutive data points) between the two groups (F3 (74–246 ms), F4 (134–228 ms), Fz (168–236 ms), C3 (90–150 ms), C4 (142–202 ms), and Cz (56–152 ms)).

The analysis of the native deviant trials showed an increase in oscillation power at theta frequency for GPs, when compared to PPs, at the F3 (74–246 ms), F4 (134–228 ms), Fz (168–236 ms), C3 (90–150 ms), C4 (142–202 ms), and Cz (56–152 ms) electrodes. Figure 1 shows the ERSP values for the theta band time-locked to the onset of the native deviant phoneme. For the other frequency ranges, no other effects were observed (except for the alpha band that increased for GPs at F3 between 82–150 ms). No differences were observed for any of the other phoneme stimulus (except for the nonnative deviant phoneme, for which one electrode - Fz - showed an increase in alpha band for PPs when compared to GPs in the 176–254 ms time range).

For the nonlinguistic conditions no significant differences were found between the groups (except for one electrode, F5, that showed an increase for PPs in theta band in the time interval 108–168 ms and in alpha band 98–228 ms).

### ITC

The analysis did not yield any significant difference between the groups for any frequency band or stimulus.

## Discussion

In the present study, we investigated the oscillatory characteristics of individual differences in the learning of the phonemes of an L2 by applying EEG spectrum analyses. The oscillatory changes related to the processing of several nonlinguistic and speech changes were compared between good and poor perceivers of an L2 speech contrast. The results of the spectral analyses showed a significant increase in the theta band power in GPs when compared to PPs in response to native speech changes at frontal and central electrodes. In line with [7], no differences between groups were found for the processing of nonlinguistic stimuli. The theta band has been repeatedly reported to be the neural oscillatory mechanism of auditory discrimination [45]–[48]. The analysis of the stimulus time-locked spectral changes revealed that GPs increased the strength of theta oscillation (ERSP) but not the intertrial coherence (ITC). Similar to the results of [7], we found differences between GPs and PPs in the theta power at frontal electrodes, but not at the temporal electrodes.

The EEG data analyzed in the present study was recorded for several tonal and speech changes in paradigms in which one stimulus type was presented frequently (standard) to create a regular context that was violated by a deviant stimulus, with a lower probability to be presented. The event-related potential response evoked by these auditory changes elicited an MMN [7]. The amplitude of the MMN was similar between GPs and PPs for the changes involving tones (nonlinguistic stimuli), but GPs showed larger MMNs for the speech changes. In the present study, when the spectral changes were analyzed, oscillations in the theta band were found to underlie the group differences in the MMN response to native phonemes. This finding is in line with previous studies relating the MMN to changes in the theta band [45], [46], [48]. As in previous studies, the stimulus time-locked power spectral changes (ERSP) in the theta band were found between 80–240 ms, the time window of the MMN. The lack of ERSP differences between the groups for the nonlinguistic stimuli converges with the similar MMNs found for the two groups in these conditions and supports the claim that PPs and GPs are similar in their skills to process auditory changes.

The analysis of the spectral modulations time-locked to the stimulus revealed that GPs and PPs differed only for the oscillation strength (ERSP), but not in the phase coherence (ITC) in the theta frequency during the MMN interval (50–250 ms) for the deviant native phoneme. We analyzed the ERSP and ITC separately for standard and deviant stimuli as previous studies [45], [46], [48] showed that the elicitation of the MMN is mainly driven by the modulation of theta oscillations for deviant stimuli. However, [47] found no evidence for event-related spectral power changes performing single trial analyses of the MMN (subtracting deviant trials from the preceding standard), but a significant phase-locking at the theta frequency. In the present study, the analysis of ERSP showed again for GPs, in comparison to PPs, an increase in theta power for the native deviant phoneme at central (C3, C4, and Cz) and frontal electrodes (F3, F4 and Fz). For the native standard phoneme, there were no differences between the two groups, suggesting that GPs and PPs process speech sounds similarly, but it is the detection of a change within the auditory context that is different between the groups. The similar pattern of neural oscillations for the unknown vowels suggests that the difference between the two groups lies in the cognitive mechanism responsible for detecting familiar speech changes, rather than the one in charge of speech acoustic analysis.

We did not observe any group difference in the responses to the unknown deviant sound in the theta frequency band. The lack of differences for the unknown phonemes differs from the results in [7]. In this previous study, the MMN elicited by the native and nonnative speech changes were analyzed by means of a single ANOVA. The analysis revealed a significant group effect, indicating that GPs showed larger MMNs than PP for both native and unknown phonemes. Despite the fact that in [7] no interaction was found between group (GP and PP) and phoneme type (native and unknown), the difference between the groups in their MMN to the unknown phoneme change was quantitatively smaller than the difference for native deviants. The present study, following the analysis procedures from previous studies on oscillatory responses underneath the MMN [45]–[48], compared the groups separately for each standard and deviant stimulus. Hence, the two groups were compared for each vowel type separately, rather than running a global analysis with the two phoneme conditions as in [7]. When the two phoneme conditions were analyzed separately, differences between the groups were only found for the native vowel. The present group differences only for the native phoneme indicate that the two groups differ mainly in the processing of familiar speech sounds. One possible explanation for this pattern is that GPs have more efficient speech processing capacities in comparison to PPs. Lifelong experience with native contrasts should result in better neural representations for GPs than for PPs, whereas the lack of previous experience with unknown sounds for all participants should diminish (if not abolish) the difference between GPs and PPs in detecting unknown contrasts.

The ERSP group differences between GPs and PPs did not concur with ITC differences. It has been shown that oscillatory phase alignment may not concur with change in power [52]. [45] found that frontal components of the MMN were formed by increases in both ITC and ERSP, whereas temporal components of the MMN were formed by phase alignment alone. However, [47] suggested that the MMN is described best by changes in the ITC. Understanding the distinction between ERSP and ITC is important for understanding the ERP generation. Whether ERPs are generated by phase-locking ongoing neural activities or they originate in additive stimulus-evoked responses is still under debate [53]–[56].

In the current study, the differences between GPs and PPs in theta oscillations were found mainly at fronto-central electrodes, whereas no difference was found at the temporal electrodes (left and right mastoids, T3L and T4L). Previous studies [45]–[48] reported the involvement of theta oscillations in both temporal and frontal areas. [45] argued that the different components (i.e. temporal and frontal) of the MMN are driven by changes in the phase alignment and power modulation, to a different extent. They found an enhanced theta ITC, but no ERSP changes at the mastoid electrodes. In contrast to the mastoid electrodes, the fronto-central electrodes showed changes of theta ERSP and ITC in the MMN intervals. These findings support the existence of different MMN sources, with distinct functional roles. Our ERSP and ITC results also showed group differences at the fronto-central electrodes, but not at the mastoid electrodes. In line with our previous findings [7], differences in speech discrimination between GPs and PPs were found at fronto-central electrodes mainly. The frontal differences between GPs and PPs suggest that the origin of individual differences in phoneme learning may be due to a functional difference of the frontal MMN generator. Hence, our analysis strengthens the conclusion that the differences between GPs and PPs may not be related to the encoding and comparison of sensory features (reflected by the temporal component of the MMN), but that they may be linked to differences in the attentive or pre-attentive detection of signal change, supported by the frontal component [30], [57].

Our results indicate the existence of differences in the theta oscillatory activity between individuals differing in their capacity to perceive foreign phonemes. The GPs showed an increased theta power and phase alignment for native speech discrimination in fronto-parietal areas, when compared to PPs. The present study provides evidence supporting the use of time-frequency analyses to understand the underlying neural mechanisms of speech processing and provides new insights into brain mechanisms involved in speech learning.
